# Exploring the therapeutic potential of modern and ancestral phenylalanine/tyrosine ammonia-lyases as supplementary treatment of hereditary tyrosinemia

**DOI:** 10.1038/s41598-020-57913-y

**Published:** 2020-01-28

**Authors:** Natalie M. Hendrikse, Albin Holmberg Larsson, Stefan Svensson Gelius, Sergei Kuprin, Erik Nordling, Per-Olof Syrén

**Affiliations:** 1 Swedish Orphan Biovitrum AB, SE-112 76 Stockholm, Sweden; 2 School of Engineering Sciences in Chemistry, Biotechnology and Health, KTH Royal Institute of Technology, Science for Life Laboratory, Tomtebodavägen 23, Box 1031, SE-171 21 Solna, Sweden; 3 School of Engineering Sciences in Chemistry, Biotechnology and Health, Department of Fibre and Polymer Technology, KTH Royal Institute of Technology, Teknikringen 56-58, SE-100 44 Stockholm, Sweden; 4 School of Engineering Sciences in Chemistry, Biotechnology and Health, Division of Protein Technology, KTH Royal Institute of Technology, Science for Life Laboratory, Tomtebodavägen 23, Box 1031, SE-171 21 Solna, Sweden

**Keywords:** Recombinant protein therapy, Metabolic disorders

## Abstract

Phenylalanine/tyrosine ammonia-lyases (PAL/TALs) have been approved by the FDA for treatment of phenylketonuria and may harbour potential for complementary treatment of hereditary tyrosinemia Type I. Herein, we explore ancestral sequence reconstruction as an enzyme engineering tool to enhance the therapeutic potential of PAL/TALs. We reconstructed putative ancestors from fungi and compared their catalytic activity and stability to two modern fungal PAL/TALs. Surprisingly, most putative ancestors could be expressed as functional tetramers in *Escherichia coli* and thus retained their ability to oligomerize. All ancestral enzymes displayed increased thermostability compared to both modern enzymes, however, the increase in thermostability was accompanied by a loss in catalytic turnover. One reconstructed ancestral enzyme in particular could be interesting for further drug development, as its ratio of specific activities is more favourable towards tyrosine and it is more thermostable than both modern enzymes. Moreover, long-term stability assessment showed that this variant retained substantially more activity after prolonged incubation at 25 °C and 37 °C, as well as an increased resistance to incubation at 60 °C. Both of these factors are indicative of an extended shelf-life of biopharmaceuticals. We believe that ancestral sequence reconstruction has potential for enhancing the properties of enzyme therapeutics, especially with respect to stability. This work further illustrates that resurrection of putative ancestral oligomeric proteins is feasible and provides insight into the extent of conservation of a functional oligomerization surface area from ancestor to modern enzyme.

## Introduction

Hereditary tyrosinemia (HT) is the collective name of rare hereditary congenital diseases that are characterised by an impaired breakdown of L-tyrosine (L-Tyr). Three types of tyrosinemia are defined to date, which are associated with impaired or depleted function of three distinct enzymes in the breakdown pathway^1^ (Supplementary Fig. S1). In HT Type I (HT1), mutations in the fumarylacetoacetate hydrolase gene result in loss of function of the corresponding enzyme (EC 3.7.1.2), effectively blocking the final step of L-Tyr catabolism. This in turn leads to the accumulation of toxic intermediates maleylacetoacetate and fumarylacetoacetate in body fluids and organs, which can induce organ failure and carcinogenesis. HT1 patients are currently treated with Nitisinone (NTBC), which inhibits an upstream enzyme (Supplementary Fig. S1) and thereby prevents the accumulation of toxic intermediates, combined with a protein-restricted diet^2^. Since NTBC terminates the pathway at an earlier stage—corresponding to the phenotype of HT Type III—L-Tyr and 4-hydroxyphenylpyruvate accumulate in plasma. It has been observed that HT1 patients treated with NTBC and a protein restricted diet are at risk of presenting developmental delay and impaired cognitive functioning, possibly due to the elevated concentration of L-Tyr^3^. Moreover, L-Phenylalanine (L-Phe) accumulates in blood plasma, as its catabolism normally proceeds *via* the L-Tyr breakdown pathway (Supplementary Fig. S1). Excess L-Phe, or hyperphenylalaninemia, is also damaging to tissues and has been associated with irreversible intellectual and developmental disabilities in connection with phenylketonuria (PKU); the genetic disease associated with impaired L-Phe breakdown^4–6^. In order to bypass the natural L-Phe/L-Tyr breakdown pathway and avoid accumulation of toxic or otherwise harmful metabolites, alternative pathways can be explored, which has previously been done for PKU^7–10^.

**Figure 1:**
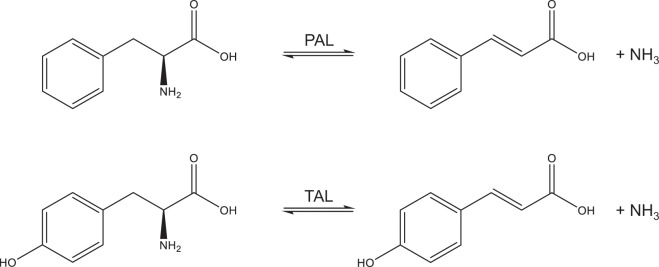
PAL and TAL catalyse the deamination of L-phenylalanine to cinnamic acid and ammonia and L-tyrosine to coumaric acid and ammonia, respectively. Charges are not shown.

Tyrosine ammonia-lyase (TAL, EC 4.3.1.23) is an enzyme that catalyses the non-oxidative deamination of L-Tyr into *p*-coumaric acid (Fig. 1) and was first identified in bacteria^11^. TAL is part of the aromatic amino acid ammonia-lyase family that also includes phenylalanine ammonia-lyase (PAL, EC 4.3.1.24), histidine ammonia-lyase (HAL, EC 4.3.1.3) and structurally and mechanistically similar class I lyase-like aminomutases^12^. The enzymes in this family are characterised by conserved residues Ala-Ser-Gly in the active site that autocatalytically form the activated group 4-methylideneimidazole-5-one^13^ (MIO) to generate a catalytically competent enzyme that is functional as a homotetramer^14^. The biocatalytic potential of this enzyme family has been explored for several applications^12,15,16^. Since both *p*-coumaric acid (Fig. 1, bottom) and cinnamic acid (Fig. 1, top) are non-toxic metabolites that are eventually excreted in urine^17,18^, TAL and PAL are potentially interesting therapeutic enzymes for treatment of HT1. In analogy, subcutaneous injection of a prokaryotic PEGylated PAL from *Anabaena variabilis* was approved by the FDA in 2018 for the treatment of PKU^19^.

Over the last two decades, several PAL enzymes that also have side activity towards L-Tyr have been identified, mostly from fungi and monocotylic plants^20,21^. These enzymes with dual activity are often referred to as PAL/TAL enzymes (EC 4.3.1.25) and some deaminate both substrates with catalytic turnovers of >1 per second^22^, which is higher than most reported activities for L-Tyr-specific TAL from bacteria (typical catalytic turnover ca. 0.1 s^−1^)^23^. Therefore, we believe that this PAL/TAL group harbours potential therapeutic enzymes that could be used for complementary treatment of HT1 in addition to NTBC, relieving the burden of a strict diet for patients as well as reducing accumulation of both L-Tyr and L-Phe. In order to enhance the therapeutic potential beyond existing PAL/TALs, in particular in terms of protein stability and relaxed substrate specificity, we turned our attention towards ancestral sequence reconstruction (ASR) as a strategy for enzyme engineering^24–26^. The benefit of using this method for enhancing properties that are beneficial for biopharmaceuticals has previously been shown for coagulation factor VIII^24^. In our group we previously explored this approach for a terpene cyclase enzyme and obtained enzyme scaffolds with increased thermostability and promiscuity^27^. Both of these properties would be of interest for a therapeutic PAL/TAL, for which prolonged half-life in the body and ability to deaminate both L-Phe and L-Tyr are of importance. Ancestral sequence reconstruction of an oligomeric protein (in this case a homotetramer) would provide insight into the performance of this method when applied to multimeric targets, a challenge which hitherto has been scarcely explored^28,29^. Moreover, our approach would also shed light on fundamental aspects of protein oligomerization that have attracted scientific interest^30,31^.

As a starting point we selected two fungal enzymes that are both reported to have relatively high L-Tyr activity: PAL/TAL from *Rhodotorula glutinis* (*Rg*PAL) and PAL/TAL from *Trichosporon cutaneum* (*Tc*PAL)^21,22^. We reconstructed several ancestral variants within their clade of the tree and evaluated their therapeutic potential in terms of activity and stability. For comparison, an extended characterization of the two modern enzymes was performed. We found that all ancestral variants had increased thermostability compared to *Rg*PAL and *Tc*PAL, albeit at the cost of lower catalytic turnover. Moreover, one ancestral variant showed considerably improved long-term stability as well as increased ability to maintain activity after incubation at 60 °C, which are both indicative of a potentially improved shelf-life. All ancestors retained the capability to oligomerize, supporting an evolutionary model that does not pass through an intermediate state with disrupted protein-protein interactions^30^. We believe that our results highlight the potential of ancestral sequence reconstruction as an approach to engineer multimeric enzymes towards therapeutic applications, especially with respect to stability.

## Results

### Reconstruction of ancestral PAL/TALs from fungi by MEGA and PAML

**Figure 2:**
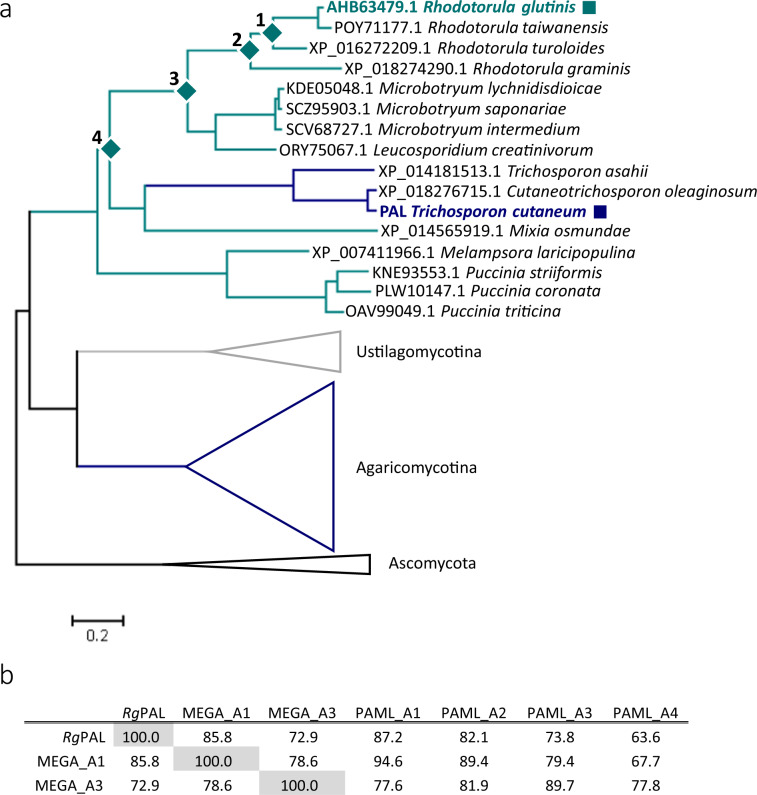
Ancestral sequence reconstruction of PAL/TAL. (**a**) Maximum Likelihood tree of PAL/TALs from fungi. The three subphyla from Basidiomycota are colored blue (Agaricomycotina), grey (Ustilagomycotina) and green (Pucciniamycotina). Sequences from Ascomycota were used as an outgroup (black). All clades apart from the Pucciniamycotina are shown as triangles, the full tree can be found in Supplementary Fig. S2. Ancestral nodes are indicated by diamonds and the two query sequences are indicated by squares. (**b)** Sequence identities shared by *Rg*PAL and the reconstructed ancestral sequences.

Due to their relatively favourable L-Phe/L-Tyr specificity ratio, *Rg*PAL and *Tc*PAL were selected as query sequences for construction of a phylogenetic tree of 68 fungal PAL/TALs (Fig. 2a, full tree in Supplementary Fig. S2). The two sequences share 50% sequence identity between them and have reported PAL/TAL specificity ratios of 2–3^22,32^. Remarkably, three sequences from subphylum Agaricomycotina—amongst which the sequence from *T. cutaneum*—cluster with the Pucciniomycotina sequences. Apart from this exception the tree topology roughly follows a species tree of Basidiomycota that was previously published^33^. We used maximum likelihood statistics implemented in PAML^34,35^ to reconstruct the most likely ancestral sequences at four different nodes (Fig. 2a). Going from young to old, A1, A2, A3 and A4 roughly span the evolutionary distance between *Rg*PAL to the last common ancestral node with *Tc*PAL. Two of the four nodes were also chosen for ancestral sequence reconstruction in MEGA7^36^ to increase our coverage of ancestral sequence space and to investigate the robustness of the reconstruction. The putative ancestral sequences differ from *Rg*PAL by 13–34% in sequence identity (Fig. 2b). Figure 2b also shows that the respective sequences of A1 and A3 from PAML and MEGA differ slightly in identity compared to *Rg*PAL: the PAML sequences are ca. 1% closer to *Rg*PAL. The two variants of the same node share sequence identities of 95% (A1) and 90% (A3), including some small indels. Inspection of the reconstructed ancestral sequences confirmed that all residues known to be catalytically important were conserved, including the Ala-Ser-Gly triad in the active site that forms the catalytic moiety MIO (Supplementary Fig. S3).

### Homology model of MEGA_A1 reveals that oligomerization interface is not conserved

**Figure 3:**
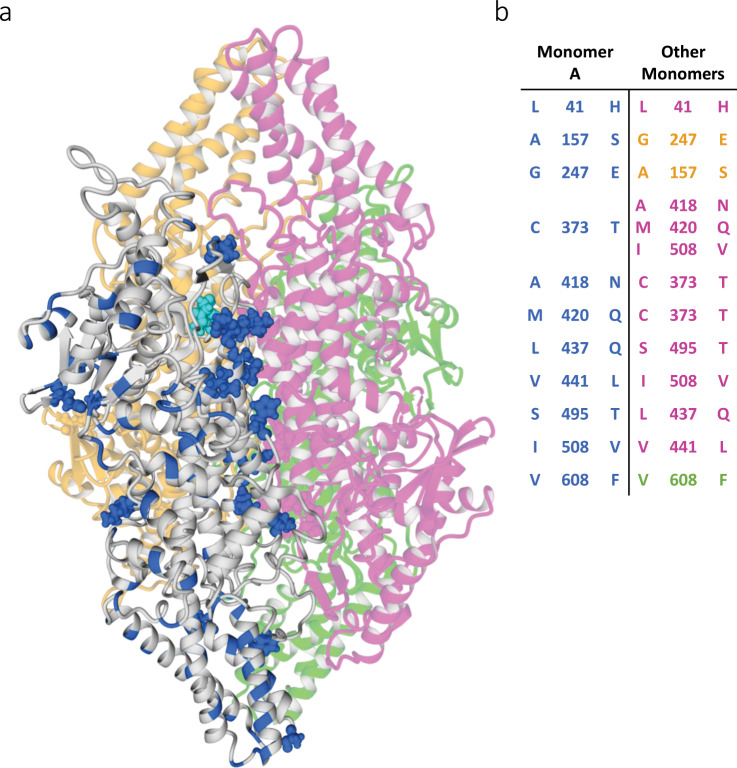
Ancestral PAL/TALs form tetramers despite non-conserved interfaces between monomers. **(a)** Homology model of MEGA_A1 showing the distribution of ancestral mutations. Monomers are coloured in grey (monomer A), magenta (monomer B), yellow (monomer C) and green (monomer D). All ancestral mutations are coloured in blue in monomer A, and surface mutations within 4 Å of another monomer are shown as balls. Catalytically active group MIO is shown in cyan for monomer A for reference of the active site location. The model was built in YASARA using the crystal structure of PAL/TAL from *Rhodotorula turoloides* (PDB, 1Y2M^7^) as a template. (**b**) Possibly coupled ancestral mutations in the oligomerization interfaces. The mutations for monomer A are listed in blue and the corresponding mutations in proximity are coloured according to monomer. Mutations are noted with *Rg*PAL residues on the left and the corresponding residue in MEGA_A1 on the right.

As the modern enzymes are active as homotetramers^21^, we were interested to see if and how the interfaces between monomers changed in the reconstruction process. To visualize the spatial distribution of ancestral mutations, a homology model was built for MEGA_A1 (Fig. 3a). We found that approximately 60% of all ancestral mutations occurred on the surface of the enzyme and that the interfaces between the monomers were not more conserved than other areas on the surface. We noted that some of the mutations in one of the monomers in the interaction surface were in proximity to ancestral mutations in the other monomer. In fact, eleven interaction surface mutations from monomer A were within 4 Å of a mutation in another monomer (Fig. 3b).

### Ancestral enzymes can be functionally expressed in *E. coli*

**Figure 4:**
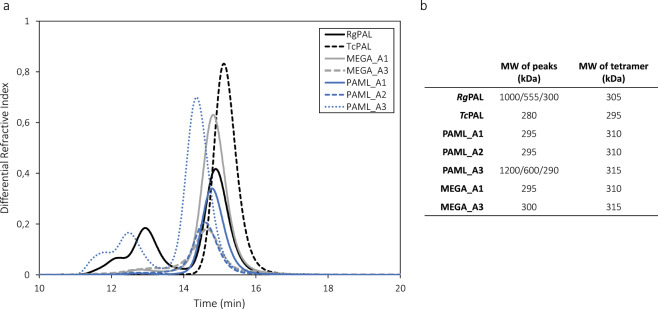
Analysis of oligomers by SEC-MALS. (**a)** Elution profiles of all enzymes from the size-exclusion chromatography column. (**b)** Molecular weights (MW) of the SEC-elution peaks as determined by MALS (left column). For comparison, the theoretically calculated MW for the respective tetramers is shown the right column. For *Rg*PAL and PAML_A3, MW is shown for all three peaks.

All enzymes except PAML_A4 could be successfully expressed in *E. coli* BL21 (DE3) and were purified by affinity chromatography followed by size exclusion chromatography. During the second step of the purification process we noted that some enzymes eluted from the size exclusion column in three separate peaks. As the active form of the enzymes has previously been found to be a homotetramer^21^, we hypothesized that these peaks may constitute different oligomeric states. To investigate this hypothesis, we applied analytical size exclusion chromatography (Fig. 4a) coupled to a multi-angle light scattering detector (SEC-MALS) to determine the approximate molecular weight of each peak eluting from the column (Fig. 4b). The results indicated that the tetramer was in fact the smallest oligomer eluting from the SEC column and that the two other peaks consisted of larger aggregates.

Interestingly, the ratio between the areas of the different peaks was not the same for all enzymes: whereas *Rg*PAL and PAML_A3 had significant peaks with higher molecular weight, *Tc*PAL, MEGA_A1, MEGA_A3, PAML_A1 and PAML_A2 appeared mainly in tetrameric form. An initial activity screen of 15 minutes was performed for all purified enzymes with both L-Phe and L-Tyr as substrates (Supplementary Fig. S4). The highest activity was detected for *Rg*PAL, *Tc*PAL and MEGA_A1, moderate activity was detected for PAML_A1 and MEGA_A3, and low activity for PAML_A2. After extended incubation for 1 hour, activity could also be detected for PAML_A3 (data not shown). Based on these results we decided to perform kinetic analysis for *Rg*PAL, *Tc*PAL, PAML_A1, PAML_A2, MEGA_A1 and MEGA_A3 to enable quantification and comparison of specific activities of these enzymes.

### Kinetic analyses show cooperativity in *Tc*PAL and reduced activity in ancestral variants

Kinetic assays were performed separately with L-Phe and L-Tyr at 37 °C (Supplementary Fig. S5). Kinetic parameters can be found in Table 1. We observed cooperativity in *Tc*PAL, characterised by the non-linear relation between reaction rate and substrate concentration at low substrate concentrations, which has also been reported previously for this enzyme^22,32^. We observed moderate cooperativity with both L-Phe and L-Tyr and determined the Hill constants to be 1.6 ± 0.03 and 1.5 ± 0.1, respectively. We found that the catalytic turnover of both substrates was higher for *Tc*PAL as compared to *Rg*PAL, yet the ratio between the PAL/TAL activities was comparable (Table 1). Kinetic assays of the ancestral variants confirmed the general trend observed in the screen: activity was reduced in nearly all ancestral variants and decreased with each step back in the tree. The two ancestors that were reconstructed in MEGA had higher activity than those reconstructed in PAML and cooperativity was not observed in any of the ancestors. We observed similar PAL/TAL ratios of catalytic turnover for all enzymes, but changes in PAL/TAL specificity ratio could be seen. The specificity ratio for both MEGA ancestors became favourable towards L-Tyr, whereas *Rg*PAL favoured L-Phe. Interestingly, both older ancestors MEGA_A3 and PAML_A2 showed substantially decreased *K*_M_ values as compared to their younger counterparts.

**Table 1:** Kinetic parameters of all enzymes for L-Phenylalanine and L-Tyrosine at 37 °C. Standard deviations are shown with n = 3.

	L-Phe^a^			L-Tyr^a^			Relative Activity^b^	
	*k*_cat_ (s^−1^)	*K*_M_^c^ (mM)	*k*_cat_/*K*_M_ (M^−1^ s^−1^)	*k*_cat_ (s^−1^)	*K*_M_^c^ (mM)	*k*_cat_/*K*_M_ (M^−1^ s^−1^)	$\frac{{[{{\boldsymbol{k}}}_{{\boldsymbol{cat}}}]}_{{\boldsymbol{Phe}}}\,}{{[{{\boldsymbol{k}}}_{{\boldsymbol{cat}}}]}_{{\boldsymbol{Tyr}}}}$	$\frac{{[{{\boldsymbol{k}}}_{{\boldsymbol{cat}}}/{{\boldsymbol{K}}}_{{\bf{M}}}]}_{{\boldsymbol{Phe}}}}{{[{{\boldsymbol{k}}}_{{\boldsymbol{cat}}}/{{\boldsymbol{K}}}_{{\bf{M}}}]}_{{\boldsymbol{Tyr}}}}$
*Rg*PAL	8.95 ± 0.37	1.35 ± 0.10	6655 ± 259	1.71 ± 0.11	0.28 ± 0.01	6303 ± 288	5.2	1.1
*Tc*PAL	13.24 ± 0.42	0.10 ± 0.03	—^c^	2.21 ± 0.28	0.07 ± 0.05	—^c^	6.0	—^c^
MEGA_A1	5.76 ± 0.25	3.83 ± 0.97	1560 ± 326	1.05 ± 0.06	0.40 ± 0.02	2643 ± 312	5.5	0.6
MEGA_A3	1.42 ± 0.03	0.35 ± 0.03	4069 ± 356	0.23 ± 0.01	0.05 ± 0.01	4509 ± 34	6.2	0.9
PAML_A1	0.30 ± 0.01	4.48 ± 0.62	68 ± 8	0.04 ± 0.01	2.69 ± 0.01	17 ± 1	6.8	4.1
PAML_A2	0.03 ± 0.01	0.69 ± 0.15	42 ± 7	—^d^	—^d^	—^d^	—^d^	—^d^

^a^Measurements were performed in a 50 mM Tris-HCl buffer at pH 9.2.

^b^Differences may occur due to rounding.

^c^For *Tc*PAL, *K’* is shown instead of *K*_M_ due to cooperativity. Hill constants were 1.6 ± 0.03 (L-Phe) and 1.5 ± 0.1 (L-Tyr).

^d^For PAML_A2, activity towards L-Tyr was not sufficient for kinetic analysis.

### Analysis of thermostability shows increased stability for all ancestors

In order to characterise the thermostability of the ancestral PAL/TAL variants we established melting curves using nanoDSF^37,38^ followed by determination of melting temperatures (Table 2, Supplementary Fig. S6). Modern enzymes *Tc*PAL and *Rg*PAL had the lowest melting temperatures of 62 °C and 66 °C, respectively. For the three ancestors reconstructed in PAML, stability increased with each step back in the tree: 71 °C (PAML_A1), 75 °C (PAML_A2), and 79 °C (PAML_A3). The melting temperature of MEGA_A1 was determined to be 71 °C, but for MEGA_A3 no clear shift in tryptophan emission wavelength could be seen (data not shown). A look at the tryptophan content of all enzymes revealed that *Rg*PAL and all PAML ancestors share the same four tryptophans, three of which also occur in the sequence of *Tc*PAL (Supplementary Fig. S3). MEGA_A1 and MEGA_A3 have 5 and 6 tryptophans, respectively, of which one in MEGA_A3 is located close to the N-terminus and may mask the signal of the others during thermal unfolding. To obtain information on the relative thermostability of MEGA_A3, thermal unfolding of the two modern enzymes and the two MEGA ancestors was also measured using CD spectroscopy (Supplementary Fig. S7). CD spectra showed that loss of secondary structure of MEGA_A1 and MEGA_A3 occurred at higher temperatures compared to both modern enzymes.

**Table 2:** Analysis of thermostability by nanoDSF. Standard deviations are shown with n = 3.

	*T*_m_ (°C)^a^
*Tc*PAL	61.7 ± 0.1
*Rg*PAL	66.2 ± 0.1
PAML_A1	71.4 ± 0.1
PAML_A2	74.8 ± 0.6
PAML_A3	78.0 ± 0.8
MEGA_A1	70.9 ± 0.1
MEGA_A3	*n.d*.

^a^Measurements were performed in a 50 mM Tris-HCl, 125 mM NaCl buffer, pH 8.5.

### Evaluation of therapeutic potential highlights increased stability of MEGA_A1 and MEGA_A3

**Figure 5:**
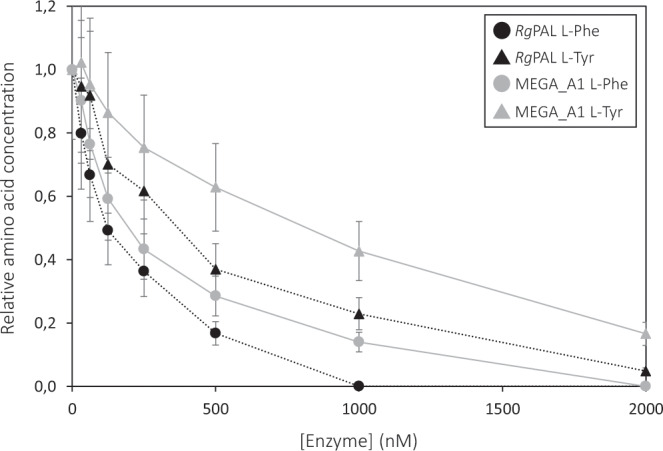
Activity of *Rg*PAL and MEGA_A1 in calf serum. Relative amino acid concentrations are shown for different enzyme concentrations. Starting concentrations were approximately 1000 µM for L-Tyr and 200 µM for L-Phe and final concentrations were determined by reversed-phase HPLC, after incubation with enzyme. Error bars shown are based on duplicates of the [E] = 0 measurement.

To investigate the potential of oral administration, proteolytic assays with two different proteases were performed for *Rg*PAL and MEGA_A1. We chose to include pepsin and trypsin; digestive enzymes from the stomach and duodenum, respectively. The reactions with pepsin were carried out at its optimum pH of 2.0 and the reactions with trypsin were carried out at pH 8.5, which is close to the optimum pH for the lyase enzymes^39^. Samples were taken at various timepoints and analysed on SDS-PAGE in addition to activity measurements with L-Tyr. Both the gel and the activity measurements showed that *Rg*PAL and MEGA_A1 were immediately inactivated by pepsin (Supplementary Fig. S8). Interestingly, the control samples without pepsin did not show enzymatic activity even though the pH had been restored to 8.5 for the activity measurements. Samples with and without trypsin were monitored over the course of 4 hours (Supplementary Fig. S8). After 4 hours both *Rg*PAL and MEGA_A1 had lost approximately 55% of their activity (Supplementary Fig. S8b) and an additional measurement after 20 hours confirmed that activity had completely disappeared. Another possible way of administration is intravenously, in which case the enzymes would need to be active in the blood. To explore this possibility, we aimed to confirm activity of MEGA_A1 and *Rg*PAL in a more physiologically relevant matrix. Calf serum was spiked with 800 µM L-Tyr to mimic levels in patients^1^ and a range of enzyme concentrations was tested to get an estimate of an appropriate dose (Fig. 5). No L-Phe was added, but approximately 200 µM was present in the serum initially. All L-Phe was cleared from samples with 2000 nM *Rg*PAL and MEGA_A1, whereas L-Tyr was reduced by 95% and 83%, respectively.

**Figure 6:**
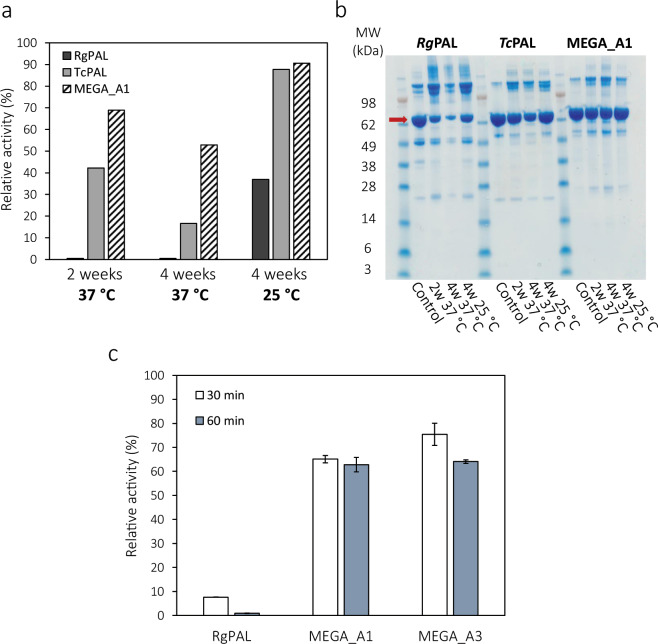
Evaluation of long-term stability. **(a)** Relative L-Phe activity of all long-term stability samples for *Rg*PAL, *Tc*PAl and MEGA_A1. Activity was measured at 37 °C and normalized to control samples that had been stored at −80 °C. L-Phe was chosen for activity evaluation based on the higher measured activity towards this substrate (Table 1). (**b)** SDS-PAGE of all samples. The SeeBlue Plus2 pre-stained molecular weight marker was used for reference and PAL enzymes are indicated by an arrow. The gel image was cropped around the edges for display. (**c)** Relative L-Phe activity of *Rg*PAL, MEGA_A1 and MEGA_A3 after incubation at 60 °C for 30 and 60 minutes. Activity was measured at 37 °C and normalized to control samples that had been stored at 25 °C. L-Phe was chosen for activity evaluation based on the higher measured activity towards this substrate (Table 1). Standard deviations are shown with n = 3.

To further explore their potential as therapeutics, samples of *Rg*PAL, *Tc*PAL and MEGA_A1 were incubated at 25 °C and 37 °C for 2–4 weeks, followed by evaluation of stability as well as L-Phe activity (Fig. 6a,b). Figure 6a shows that MEGA_A1 samples were most active in all three cases and that up to 52% of activity was retained after 4 weeks at 37 °C. Even *Tc*PAL was significantly more stable than *Rg*PAL, which lost activity completely after 2 weeks at 37 °C. Analysis on SDS-PAGE (Fig. 6b) supports these findings as the band around 75 kDa becomes fainter in *Rg*PAL samples and slightly fainter in *Tc*PAL samples, but not for MEGA_A1. All three enzymes seemed more tolerant to 4 weeks at 25 °C than 2 weeks at 37 °C. We were also interested in testing heat resistance of the most active ancestral variants, MEGA_A1 and MEGA_A3 in comparison to *Rg*PAL, the modern variant displaying the highest *T*_m_ (Table 2). Figure 6c shows the relative activity of the three enzymes after incubation at 60 °C for 30 and 60 minutes, as compared to a control not subjected to heat. Whereas *Rg*PAL lost nearly all activity after 60 minutes, MEGA_A1 and MEGA_A3 retained 63% and 64% of their activity, respectively.

## Discussion

In this study we aimed to explore if ancestral sequence reconstruction could be a viable strategy to enhance therapeutic properties of phenylalanine/tyrosine ammonia-lyases from fungi, towards a complementary treatment of hereditary tyrosinemia. We found that all ancestral variants showed increased thermostability, albeit at the expense of catalytic turnover as compared to modern enzymes *Rg*PAL and *Tc*PAL. MEGA_A1 displayed substantially enhanced long-term stability at 25 and 37 °C and, together with MEGA_A3, significantly increased resistance to incubation at 60 °C compared to *Rg*PAL, which highlights the possibilities of ancestral sequence reconstruction for engineering stable oligomeric therapeutic enzymes.

It has previously been reported that fungal PAL is monophyletic, *i.e*. that all genes derive from a common ancestral gene that was present in the ancestor of the subkingdom Dikarya or earlier^40^. We used two different programs—MEGA and PAML—for ancestral reconstruction of PAL/TALs at several nodes within the Pucciniomycotina, a subphylum of the division Basidiomycota. The divergence of the three subphyla in this division is estimated to have occurred between 1030 and 670 million years ago, but remains ambiguous^41^, which makes it difficult to estimate an age for the ancestral enzymes. Interestingly, a group of three sequences from the Agaricomycotina was placed within the clade of Pucciniomycotina with high support values, in trees built with Maximum Likelihood as well as Bayesian Inference (Supplementary Figs. S2 and S9). Amongst these was the query sequence from *T. cutaneum* that, in contrast to the other characterised enzymes, displays cooperativity in kinetic analyses^22,32^. This tree topology and the fact that it is the only enzyme displaying cooperative kinetics could indicate that a horizontal gene transfer took place between the two subphyla, which would mean that node 4 is not a true divergence point in evolution. In that case node 4 does not represent an actual common ancestor of the sequences in that clade, which could be a possible explanation as to why PAML_A4 cannot be functionally expressed^42^. In this study we used two different methods to reconstruct ancestral sequences that are both based on maximum likelihood statistics^34,36^. Despite the relatively small differences between the sequences from MEGA and PAML—5% at node 1 and 10% at node 3—differences in activity of up to two orders of magnitude were observed. As all residues known to be catalytically relevant are conserved, there is no obvious sequence-based explanation for the lower activity of the PAML variants. Most differences, including some small insertions in both sequences, occur close to the N-terminal, which is highly variable in all sequences (Supplementary Fig. S3). Moreover, the knowledge regarding the catalytic mechanism of the enzyme family remains ambiguous^12,43,44^, making it highly challenging to understand the influence of point mutations from a structural perspective.

Apart from the generally reduced activity of the ancestral enzymes, we observed a decrease in *k*_cat_ accompanied by a decrease in *K*_M_ for both MEGA and PAML ancestors when going back in the phylogenetic tree. This finding could be in accordance with the previously proposed stability-activity trade-off^45^. As we envisioned the use of this enzyme family for complementary treatment of tyrosinemia, we were particularly interested in turnover of L-Tyr and the ratio between PAL/TAL activity. In terms of *k*_cat_ we found similar ratios for all enzymes, namely between 5.2 and 6.8, with the lowest being *Rg*PAL followed by MEGA_A1 (5.5). Looking at the catalytic efficiencies, Table 1 shows that both MEGA ancestors have a favourable specificity towards L-Tyr, whereas *Rg*PAL favours L-Phe. *K*_M_ values for L-Tyr are around 300–400 µM for *Rg*PAL and MEGA_A1 and *K’* for *Tc*PAL is 70 µM. These values are below typical L-Tyr blood levels in patients with tyrosinemia^1^, which means that a therapeutic enzyme would be active under *k*_cat_-conditions. In terms of *k*_cat_ for L-Tyr, *Rg*PAL and *Tc*PAL have values of around 2 s^−1^, whereas MEGA_A1 has a *k*_cat_ of 1 s^−1^. This difference of approximately 1 s^−1^ is less than the absolute differences for L-Phe turnover and its effect on dosage in patients could be compensated for by the increase in long-term stability. Activity measurements in plasma for *Rg*PAL and MEGA_A1 confirm that both enzymes successfully lower L-Tyr levels, despite the sub-optimal pH. Interestingly, MEGA_A3 has a slightly less favourable relative specificity towards L-Tyr than MEGA_A1. However, both modern enzymes in this study are also promiscuous and the evolution of different specificities among the family of aromatic amino acid ammonia-lyases remains unclear^40^.

With respect to the oligomerization state of the enzymes, we found the tetramer to be the main form in solution for all enzymes. Interestingly, analytical SEC elution profiles of *Rg*PAL and PAML_A3 also contained two peaks that seemed to constitute of multitudes of tetramers (2–4) based on the estimated molecular weight (Fig. 4). Similar peak distributions were observed in the SEC purification profile (data not shown). The fact that the ancestral mutations are scattered over the enzyme (Fig. 3a) as well as the lack of knowledge about where on their surface the tetramers interact makes it difficult to pinpoint which residues could influence the interaction. Moreover, a large percentage of the mutations in PAML_A3 are shared with PAML_A2 and PAML_A1, both of which only display one major peak in the SEC elution profile. In the context of possible therapeutic applications of these enzymes, the tendency of tetramers to aggregate is clearly a drawback due to an increased risk of immunogenicity^46^. This problem has previously also been described for PAL from *Anabaena variabilis* (the PAL variant approved for treatment of PKU) and was overcome by mutation of two cysteine residues^47,48^. Another observation regarding the oligomerization was that the interaction interface in MEGA_A1 was not more conserved than the remaining surface area. However, it has previously been shown that oligomeric interaction is not only—or not necessarily—determined by mutations in the actual interface, but also through long-range effects within the subunits^28,49^. Moreover, eleven ancestral surface mutations in one monomer were accompanied by an ancestral mutation in another monomer within a 4 Å distance (Fig. 3). Some of these mutations could be coupled, which might be accounted for by the reconstruction method since the enzyme is a homotetramer and each subunit contains the same mutations. Formation of larger oligomers than tetramers was reduced for MEGA_A1 compared to *Rg*PAL, which further enhances the therapeutic potential of this variant, despite the decrease in catalytic turnover. Another bioinformatics method often found to increase stability of proteins is the consensus method. However, this approach entails difficulties in identifying correlated mutations as those observed in the interface region^50^. These findings suggest that ancestral sequence reconstruction can also successfully be applied in the context of oligomeric proteins, which is of interest as many biopharmaceutical targets are of complex nature.

In their study, Wang *et al*. also identify the need to improve thermal stability of ammonia-lyase enzymes for therapeutic applications^48^. We found that all ancestral enzymes were more thermostable than *Rg*PAL and *Tc*PAL, with melting temperatures above 70 °C. Perhaps more importantly, MEGA_A1 displayed a substantially increased long-term stability, both at 25 °C and 37 °C. Whereas *Rg*PAL lost all its activity after 2 weeks at 37 °C, MEGA_A1 retained more than 50% of its activity after 4 weeks at 37 °C. In contrast, *Tc*PAL retained 16% of its activity after 4 weeks at 37 °C. These results show a considerable improvement in therapeutic potential for MEGA_A1, as long-term stability directly translates to shelf-life of a drug. Moreover, an increased stability at 37 °C is highly relevant from a therapeutic perspective, as it reflects *in vivo* conditions under which a potential therapeutic protein would act. Molecular mechanisms of thermostability have been discussed in the literature^51,52^. Overall, the 109 amino acid substitutions in MEGA_A1 compared to *Rg*PAL resulted in an increased number of polar residues, indicating a decrease in hydrophobicity. It is worth to note that the ancestral substitutions suggest a decreased flexibility of MEGA_A1, as the number of glycine residues decreased while the proline content increased (Pro:Gly, *Rg*PAL 27:52; MEGA_A1 30:48). We found that MEGA_A1 and MEGA_A3 displayed increased resistance to incubation at 60 °C compared to *Rg*PAL. Whereas *Rg*PAL lost nearly all activity after incubation for 60 minutes, both ancestral variants retained >60% of their activity. The introduction of proline residues into flexible loops has been shown to increase the thermostability of proteins and contribute to backbone rigidity^53,54^. For MEGA_A1 the number of prolines was increased by 3 compared to *Rg*PAL, 2 of which are located in loop regions and thus might be able to confer the observed stability increase. As for MEGA_A3, the sequence contains 12 more proline residues as compared to *Rg*PAL and a similar reasoning could be applied there.

Several ways of administration of the therapeutic enzyme could be envisioned, such as oral administration, subcutaneous or intravenous injection. Whereas the former would be aimed at clearing L-Phe and L-Tyr in the gastrointestinal tract before it is absorbed, the latter would reduce blood plasma levels of the amino acids. Both *Rg*PAL and MEGA_A1 were susceptible to degradation by pepsin and trypsin, as well as inactivation by low pH (2.0). Moreover, other studies have shown that *Rg*PAL is also highly susceptible to cleavage by other proteases such as chymotrypsin^55^. Given that oral administration would expose the enzyme to all of the above unless some form of coating is applied^56^, it seems likely that higher *in vivo* activity can be achieved through injection. The pH in serum is approximately 7.5, which is slightly below the optimum pH for PAL/TAL^39^. However, we have shown that both *Rg*PAL and MEGA_A1 are active in calf serum and decrease both L-Phe and L-Tyr levels, indicating the potential of intravenous administration.

In summary, we obtained ancestral PAL/TAL variants with decreased catalytic turnover, but considerably enhanced stability as compared to two modern fungal enzymes, not only with higher melting temperatures but also promising long-term stability at 37 °C and increased resistance to heat inactivation (as compared to *Rg*PAL). Overall, the ancestral variants displayed properties that could make them potential scaffolds amenable for further engineering towards complementary treatment of tyrosinemia. The ancestral enzymes retained their oligomeric properties even though the interface regions were not more conserved than the exposed surface area, indicating that ancestral sequence reconstruction is feasible for multimeric proteins that are of importance from a biopharmaceutical perspective.

## Methods

### Ancestral sequence reconstruction and homology modeling

A protein BLAST search was performed via NCBI in the non-redundant database using the sequence of PAL from *Rhodotorula glutinis* (GenBank accession no. AHB63479) as a query. A second BLAST search was performed using the sequence of PAL from *Trichosporon cutaneum*, which was taken from patent US007572612B2^57^. The respective 150 closest eukaryotic homologues were selected for further investigation. Duplicates, incomplete sequences, recombinant proteins and mutants were removed, before aligning the sequences using the L-INS-i algorithm in MAFFT version 7^58,59^. The sequences were trimmed using trimAl^60^ and the implemented “gappyout” method. Both IQ-TREE and MEGA found the LG model^61^ +F to be the best model for the dataset, including a gamma distribution (4 categories) to model rate variation across sites. A maximum likelihood tree was then constructed in IQ-TREE^62^ with 1000 bootstrap replicates. Based on the topology of the initial tree, the sequence selection was manually adjusted to generate a selection that covered all three phyla within Basidiomycota. In order to assess robustness of the tree, three independent Markov Chain Monte Carlo chains were run in BAli-Phy version 3.3^63^ with the same set of sequences, yet in full length. BAli-Phy simultaneously estimates the multiple sequence alignment and phylogenetic tree using Bayesian statistics. The consensus tree from the three runs is shown in Supplementary Fig. S9 and shows that only two minor differences occur with respect to the ML tree - all within the clade of Agaricomycotina. Especially the part in which the ancestors were reconstructed has high posterior probabilities. We inferred the most probable ancestral sequences at four different nodes in PAML version 4^35^, going back from *Rg*PAL to the last node shared with *Tc*PAL. In order to increase our sampling of ancestral sequence space we also inferred the most probable ancestral sequences at node 1 and 3 in MEGA version 7^36^. The homology model of MEGA_A1 was built in YASARA^64^ (version 18.4) using the crystal structure of PAL/TAL from *Rhodotorula turoloides* (1Y2M^7^) as a template. Loop regions that were missing from the crystal structure were sampled from the PDB for possible conformations and were finally optimized in YASARA using a YASARA force field. The Z-score of the resulting model was -0.495.

### Protein expression and purification

All sequences were codon-optimized for expression in *E. coli* and synthesized by GeneArt (ThermoFisher Scientific) with an N-terminal 6xHis-tag for affinity purification. The genes were subcloned into a pET22b-(+) vector (Novagen, Merck Millipore) using the NdeI and XhoI restriction sites and transformed into *E. coli* BL21 (DE3) cells using a heat-shock protocol. A single colony from each construct was inoculated in 10 mL TB medium containing 100 μg/mL ampicillin and incubated at 160 rpm at 37 °C overnight. The overnight cultures were diluted to an OD_600_ of approximately 0.07 in 40 (ancestors and *Tc*PAL) and 400 mL (*Rg*PAL) TB medium containing 100 μg/mL ampicillin, and were incubated at 160 rpm at 37 °C. The cultures were induced with 0.4 mM isopropyl β-D-1-thiogalactopyranoside (IPTG) at OD_600_ ≈ 0.5–0.7 and expression was performed for 22 hours at 160 rpm at 25 °C. Cells were harvested by centrifugation for 15 min (4 °C, 2264 x g) and the supernatant was discarded. For purification, cell pellets were resuspended in 30 ml resuspension buffer (25 mM Tris-HCl, 500 mM NaCl, 20 mM Imidazole, pH 8.5). The mixtures were sonicated on ice for 2 × 45 s effective time (5 s on 5 s off at 100% amplitude, Sonics & Materials), and centrifuged for 30 min at 4 °C, 48298 x g (Beckman Coulter). The supernatant was filtered through a 0.22 μm filter, before being loaded onto a 1 ml HisTrapHP Ni-Sepharose column installed in an ÄKTA Explorer (GE Healthcare). Protein was eluted with elution buffer (25 mM Tris-HCl, 500 mM NaCl, 500 mM Imidazole, pH 8.5) and elution fractions were analyzed on SDS-PAGE. Fractions containing the correctly sized protein were pooled, concentrated, and loaded onto a 120 ml HighLoad 16/60 Superdex200 column (GE Healthcare) which was equilibrated with SEC buffer (50 mM Tris-HCl, 125 mM NaCl, pH 8.5). A flow rate of 1.5 ml/min was used. Elution fractions were analyzed on SDS-PAGE, and fractions containing protein of the correct size were pooled, concentrated and stored at -80 °C.

### Analytical size exclusion chromatography

All purified enzymes were diluted to final concentrations of ca. 1–2 mg/ml in SEC buffer. Samples of 50 μl were loaded onto a Superdex200 Increase 10/300 GL column, which was equilibrated with filtered PBS (pH 7.4) and coupled to an ÄKTAmicro (GE Healthcare). Proteins were eluted with a flow rate of 0.75 ml/min using filtered PBS (pH 7.4), and molecular weight of the proteins in the elution peaks was determined through MALS detection, using a miniDAWN detector (Wyatt Technology). The column was calibrated using low and high molecular weight gel filtration calibration kits (GE Healthcare).

### Activity assays and kinetic analyses

Kinetics assays were performed in triplicates at 37 °C. Reactions were carried out in a 50 mM Tris-HCl buffer at pH 9.2 in a total volume of 200 μL. Substrates were obtained in pure form from Sigma-Aldrich, the final substrate concentrations in the kinetics assay were 44 µM-45 mM (L-Phe) 35 µM–9 mM (L-Tyr) and the enzyme concentrations were 0.3–0.5 µM. Formation of *trans*-cinnamic acid and *p*-coumaric acid was followed spectrophotometrically by monitoring absorbance at 290 nm and 310 nm, respectively. Extinction coefficients of 9116 M^−1^ cm^−1^ (*trans*-cinnamic acid) and 11558 M^−1^ cm^−1^ (*p-*coumaric acid) were determined in the assay buffer and were used to calculate product concentrations according to Lambert Beers law. Kinetic parameters *K*_M_ and *k*_cat_ were obtained by fitting the Michaelis-Menten equation to a plot of the reaction rates versus substrate concentrations. For *Tc*PAL, the hill equation was used instead.

### Thermostability measurements

Thermostability measurements were performed on a Prometheus NT.Plex nanoDSF instrument (NanoTemper Technologies) in a 50 mM Tris-HCl, 125 mM NaCl buffer (pH 8.5). Protein unfolding was monitored by following the ratio of intrinsic protein fluorescence at 350 nm to 330 nm over time, increasing the temperature from 20 °C to 95 °C with 1 °C per minute. The melting temperature corresponds to the maximum of the first derivative of the 350/330 ratio. CD spectroscopy measurements were performed on a Chirascan-plus (Applied Photophysics) in a 10 mM Na_2_HPO_4_/NaH_2_PO_4_ buffer (pH 8.5) using l = 0.1 cm, 0.3 mL quartz cuvettes. A temperature ramp was set during which the temperature was increased with 0.5 °C per minute from 20 °C to 90 °C, and CD spectra were obtained between 200–260 nm at 1 °C intervals.

### Incubation at 60 °C and activity measurements

For the prolonged 60 °C incubation, the enzymes were placed in a Thermomixer comfort (Eppendorf) at 60 °C for 30 and 60 minutes and were cooled to 37 °C before starting kinetic activity measurements. Enzymes that had been stored in 25 °C during the incubation were pre-heated to 37 °C for 15 minutes, as a reference for activity. Reactions were carried out in triplicates, in a total volume of 200 µL at 37 °C with L-Phe as substrate (final concentration 4.5 mM). The final enzyme concentrations were between 0.4–0.5 µM. Absorbance was measured spectrophotometrically at 290 nm.

### Assessment of proteolytic stability

To assess susceptibility to pepsin cleavage, reactions of 150 μL were set up with 67 μg/ml pepsin (from pig gastric mucosa, Roche) dissolved in 10 mM HCl at pH 2.0 and 0.4 μM (≈32 μg/ml) of *Rg*PAL or MEGA_A1, respectively. As a control, the enzymes were dissolved in 150 μL 10 mM HCl without pepsin at the same concentration. Reactions were carried out in 96-well plates at 37 °C and samples were taken at t = 0, 15, 35, 60, 90 and 120 min. A part of the sample was then used for SDS-PAGE analysis and a part was used to measure activity for 5 minutes with 1 mM L-Tyr at 37 °C. To assess susceptibility to trypsin cleavage, reactions of 600 μL were set up with 10 μg/ml trypsin (Sequencing Grade Modified Trypsin, Promega) dissolved in 50 mM Tris-HCl, 125 mM NaCl buffer at pH 8.5 and 1 μM (≈80 μg/ml) of *Rg*PAL or MEGA_A1, respectively. As a control, the enzymes were dissolved in 600 μL buffer without trypsin at the same concentration. Reactions were placed at 37 °C at 600 rpm and samples were taken at t = 12, 24, 36, 50, 80, 120, 160 and 240 min. At each time point, the sample was taken and immediately mixed with 1 mg/ml trypsin inhibitor (Aprotinin from bovine lung, Sigma-Aldrich) to quench the reaction (a ratio of inhibitor to trypsin of ca. 6:1). A part of the sample was then used for SDS-PAGE analysis and a part was used to measure activity for 5 minutes with 700 μM L-Tyr at 37 °C. To ensure that activity was completely lost in the samples with trypsin, a final sample was taken 20 hours after starting the reactions.

### Activity measurements in serum

Enzymatic breakdown of L-Tyr in calf serum was measured at different concentrations of *Rg*PAL and MEGA_A1. Calf serum was spiked with 800 μM of L-Tyr. Enzyme solutions in a range of concentrations were added to the serum, resulting in a final reaction volume of 500 μL and a final L-Tyr concentration of 775 μM. The final enzyme concentrations were 2000, 1000, 500, 250, 125, 62.5, 31.25 and 0 nM. Reactions were carried out in a thermoshaker at 37 °C and 600 rpm. The vials with *Rg*PAL were frozen at -80 °C after 90 minutes and the reactions with MEGA_A1 were frozen after 120 minutes. Samples were analyzed by RP-HPLC chromatography applying a sodium acetate/acetonitrile gradient. Pre-column derivatization with 6-aminoquinolyl-N-hydroxysuccinimidyl carbamate (AQC) was employed and subsequent detection was done by UV absorbance at 254 nm.

### Long-term stability assessment

For assessment of long-term stability of *Rg*PAL, *Tc*PAL and MEGA_A1, aliquots of 200 μL were prepared with 1 mg/ml of enzyme in SEC buffer (50 mM Tris-HCl, 125 mM NaCl, pH 8.5). Samples were filtered using 0,22 μm filters under sterile conditions and were incubated at either 25 °C or 37 °C. One aliquot of each enzyme was stored at -80 °C for reference. After 2 weeks, one sample per enzyme was withdrawn from 37 °C and stored at -80 °C. After 4 weeks additional samples were withdrawn from 25 °C and 37 °C and stored at -80 °C. All samples were thawed simultaneously and analyzed using SDS-PAGE and activity assays at 37 °C.

## Supplementary information

Supplementary Information.

## Data Availability

Additional data is available in the Supplementary Information file.
