# Supplementary material for: Exploring the therapeutic potential of modern and ancestral phenylalanine/tyrosine ammonia-lyases as supplementary treatment of hereditary tyrosinemia
Source: Sci Rep. 2020 Jan 28;10:1315. doi: 10.1038/s41598-020-57913-y (PMC6987202; doi:10.1038/s41598-020-57913-y)
Supplement: Supplementary file 1 — Supplementary Information. [file 41598_2020_57913_MOESM1_ESM.pdf]

SUPPLEMENTARY INFORMATION

Exploring the therapeutic potential of modern and ancestral phenylalanine/tyrosine ammonia-lyases as supplementary treatment of hereditary tyrosinemia

Natalie M. Hendrikse[1,2,3], Albin Holmberg Larsson[1,2], Stefan Svensson Gelius[1], Sergei Kuprin[1], Erik Nordling[1] & Per-Olof Syrén[2,3,4*]

[1] Swedish Orphan Biovitrum AB, SE-112 76 Stockholm, Sweden.

[2] School of Engineering Sciences in Chemistry, Biotechnology and Health, KTH Royal Institute of Technology, Science for Life Laboratory, Tomtebodavägen 23, Box 1031, SE-171 21 Solna, Stockholm, Sweden.

[3] School of Engineering Sciences in Chemistry, Biotechnology and Health, Department of Fibre and Polymer Technology, KTH Royal Institute of Technology, Teknikringen 56-58, SE-100 44 Stockholm, Sweden.

[4] School of Engineering Sciences in Chemistry, Biotechnology and Health, Division of Protein Technology, KTH Royal Institute of Technology, Science for Life Laboratory, Tomtebodavägen 23, Box 1031, SE-171 21 Solna, Stockholm, Sweden.

[*] Corresponding author:

Per-Olof Syrén
per-olof.syren@biotech.kth.se
Phone: +46-8-7906508

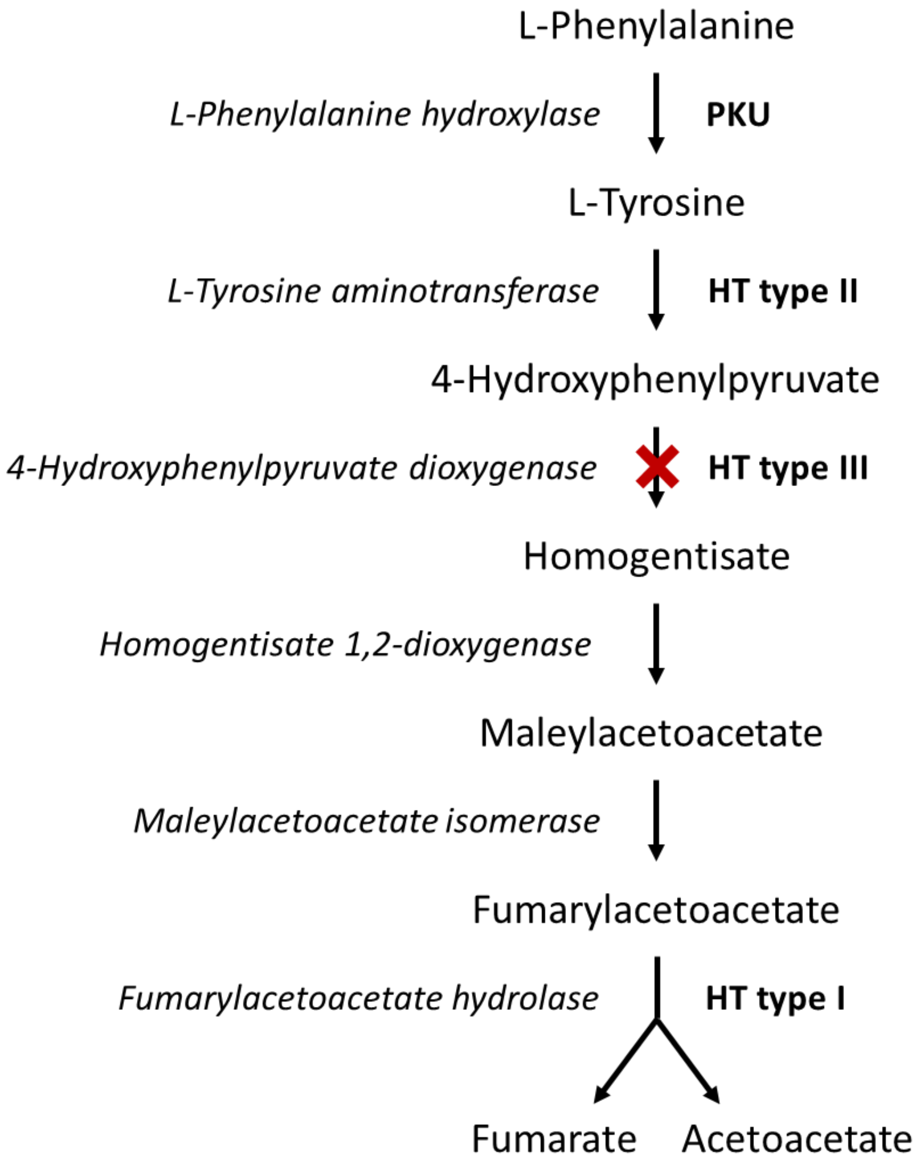

Supplementary Figure S1: L-Phenylalanine and L-Tyrosine catabolism. The enzyme responsible for each step (indicated by an arrow) is shown in italics on the left and the corresponding inborn error of metabolism is shown in bold on the right. The step blocked by Nitisinone is indicated by a red cross[1].

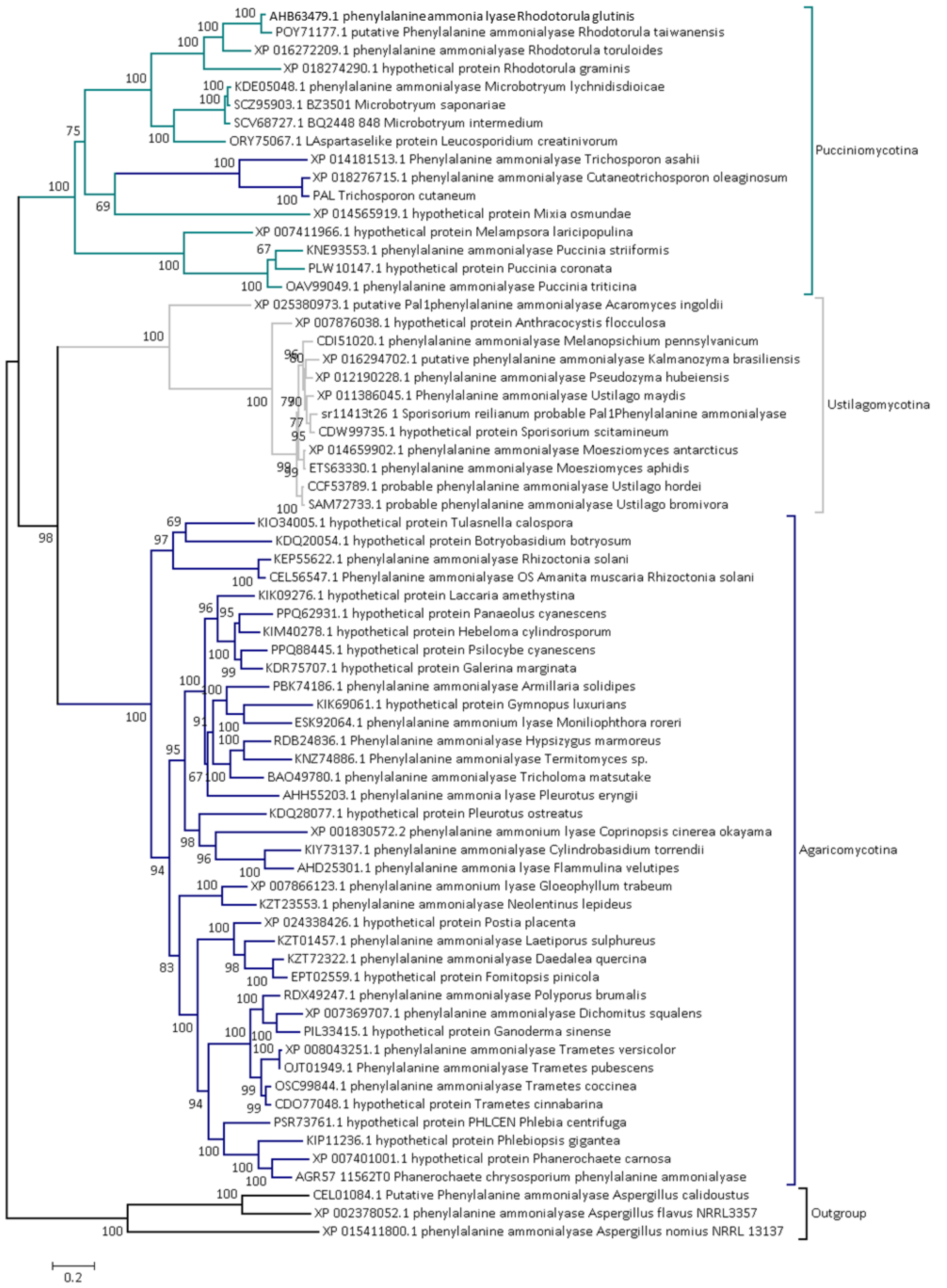

Supplementary Figure S2: Phylogenetic tree of PAL/TAL from fungi. The top part in colour consist of sequences from Basidiomycota and the bottom part in black consists of sequences from Ascomycota, which were used as outgroup to root the tree. Branches within the Basidiomycota are coloured according to subdivision: Agaricomycotina (blue), Ustilagomycotina (grey) and Pucciniomycotina (green). Bootstrap values shown are based on 1000 replicates.

```
RgPAL            MAPSVDSIATSVANSLSNGL----HAAAAANGGDVHKKTAGAGSLLPTTE
MEGA_A1          MAPSLDSISHSFANGVPNGF----HAKQAANGASTHKKTAVAGSHLPTTE
MEGA_A3          MAPSADSFTHSLTNPVPNWFGHTPRAAAAANGP--KKTPPLSPSLGPTTL
PAML_A1          MAPSLDSIAHSLANSVSSGAGHL-NAAAAANGA--SKPTALSGLLLPTTE
PAML_A2          MAPSLDSFADSLTHSVPSFAGHL-NATAAANGP--HKPTALSGLLGPTTE
PAML_A3          MAPSADSFPDSLTHSVPSFAGHL-NAAAAASGP--SPSRELSGLLAPTTL
PAML_A4          MAPSADSFPDSLTHGVPSFAGHR-SAAAAANGP--ASSREMSGLLARTTL
                 **** **:. *.:: :..       *  **.*         :     **

RgPAL            TTQLDIVERILADAGATDQIKLDGYTLTLGDVVGAARRGRSVKVADSPHI
MEGA_A1          TTQVDIVEKMLADAPTTDTIELDGYSLTLGDVVSAARKGRPVKVKDSPDI
MEGA_A3          TDQVDLLERMSADATNADTIKLDGYSLTLGDVVAAARKGAKVKIDDDPEI
PAML_A1          TTQVDIVERILADASTTDTIELDGYSLTLGDVVSAARKGRSVKVKDSPDI
PAML_A2          TTQVDIVERIAADATTADTIELDGYSLTLGDVVSAARKGRKVKIKDDPDI
PAML_A3          TSQVDLLERLAADATSADTIKLDGYSLTLGDVVAAARKGAKVKIDDDPAI
PAML_A4          TSKVEALERLASDATNSKTITLDGHSLTVGDVVAAARYGAKVKIDDDPAI
                 * ::: :*:: :**  :. * ***::**:****.*** *  **: *.* *

RgPAL            REKIDASVEFLRTQLD-NSVYGVTTGFGGSADTRTEDAISLQKALLEHQL
MEGA_A1          REKIDKSVEFLRSQLD-NSVYGVTTGFGGSADTRTEDAISLQKALLEHQL
MEGA_A3          KKRIDESVEFLKSKLD-NSVYGVTTGFGGSADTRTQDPLALQIALLEHQL
PAML_A1          REKIDKSVEFLRSQLDGNSVYGVTTGFGGSADTRTEDAISLQKALLEHQL
PAML_A2          RERIDESVEFLRSQLDGNSVYGVTTGFGGSADTRTEDAISLQKALLEHQL
PAML_A3          RKRIDESVEFLKSKLNGNSVYGVTTGFGGSADTRTEDPLALQIALLEHQL
PAML_A4          RKRIEESVEFLESKLNGTSIYGVTTGFGGSADTRTNDPAALQIALLEHQL
                 :::*: *****.::*: .*:***************:*. :** *******

RgPAL            CGVLPTSMDGFALGRGLENSLPLEVVRGAMTIRVNSLTRGHSAVRIVVLE
MEGA_A1          CGVLPTSFDSFSLGRGLENSLPLEVVRGAMTIRVNSLTRGHSAVRLVVLE
MEGA_A3          CGVLPTSFDSFALGRGLENAMPLEVVRGAMLIRVNSLTRGHSAVRLEVLE
PAML_A1          CGVLPTPLASFSLGRGLENSLPLEVVRGAMTIRVNSLTRGHSAVRLVVLE
PAML_A2          CGVLPTPLASFSLGRGLENAMPLEVVRGAMLIRVNSLTRGHSAVRLVVLE
PAML_A3          CGVLPTSSSSFSLGRGLENAMPLEVVRGAMLIRVNSLTRGHSAVRLEVLE
PAML_A4          CGVLPTSSSAFSLSDPLSNAMPEPIVRGAMLIRVNSLVRGHSAVRLEVLE
                 ******.  .*:*.  *.*::*  :***** ******.*******: ***

RgPAL            ALTNFLNHGITPIVPLRGTISASGDLSPLSYIAASITGHPDSKVHV--DG
MEGA_A1          ALTNFLNHGITPIVPLRGTISASGDLSPLSYIAAAITGHPDSKVHVVHEG
MEGA_A3          TLVKFLNHGITPIVPLRGSISASGDLSPLSYIAGAITGHPDIKVFTCIDG
PAML_A1          ALTNFLNHGITPIVPLRGTISASGDLSPLSYIAAAITGHPDSKVHVVHEG
PAML_A2          ALVNFLNHGITPIVPLRGSISASGDLSPLSYIAAAITGHPDSKVHVVQDG
PAML_A3          TLVNFLNHGITPVVPLRGSISASGDLSPLSYIAGAITGHPDIKVFVVDGL
PAML_A4          TLVNFLNHGITPVVPLRGSISASGDLSPLSYIAGAICGHPDIRVFAVDGL
                 :*.:********:*****:**************.:* **** :*..

RgPAL            --KIMSAQEAIALKGLQPVVLGPKEGLGLVNGTAVSASMATLALTDAHVL
MEGA_A1          KEKIMSAREAMALYGLEPVVLGPKEGLGLVNGTAVSASMATLALHDAHML
MEGA_A3          KEEIMPAPEALKLHGIEPVVLGPKEGLGLVNGTAVSASMATLALHDSHML
PAML_A1          KEKIMSAREALALYGLEPVVLGPKEGLGLVNGTAVSASMATLALHDAHML
PAML_A2          KEKIMPAREALALYGLEPVVLGPKEGLGLVNGTAVSASMATLALHDAHML
PAML_A3          KEEIMPAPEALKLHGIEPVVLGPKEGLGLVNGTAVSASMATLALHDAHML
PAML_A4          KKQIMPAPEALKKHGIEPIVLGPKEGLGLVNGTAFSASAASLALHDAHML
                   :**.* **:   *::*:***************.*** *:*** *:*:*
```

```
RgPAL           SLLAQALTALTVEAMVGHAGSFHPFLHDVTRPHPTQIEVARNIRTLLEGS
MEGA_A1         SLLSQALTAMTVEAMVGHAGSFHPFLHDVTRPHPTQIEVARNIRTLLEGS
MEGA_A3         ALLSQALTAMTVEAMVGHAGSFHPFIHDVCRPHPGQIEVARNIRTMLEGS
PAML_A1         SLLAQALTAMTVEAMVGHAGSFHPFLHDVTRPHPTQIEVARNIRTLLEGS
PAML_A2         ALLAQALTAMTVEAMVGHAGSFHPFIHDVTRPHPGQIEVARNIRTLLEGS
PAML_A3         ALLSQALTAMTVEAMVGHAGSFHPFIHDVCRPHPGQVEVARNIRTLLEGS
PAML_A4         ALLSQVLTAMTVEAMVGHAGSFHPFIHDVCRPHPGQVEVARNIRNMLEGS
                :**:*.***:***************:*** **** *:*******.:****

RgPAL           KYAVHHETEVKVKDDEGILRQDRYPLRCSPQWLGPLVSDMIHAHAVLSLE
MEGA_A1         KFAVHHEEEVKVKDDEGILRQDRYPLRTSPQWLGPLVSDLIHAHAVLSIE
MEGA_A3         KFAVHHEEEVKVDEDEGILRQDRYPLRTSPQWIGPLVEDLISAHRALSIE
PAML_A1         KFAVHHEEEVSQKDDEGILRQDRYPLRTSPQWLGPLVSDLIHAHAVLSIE
PAML_A2         KFAVHHEEEVSQKDDEGILRQDRYPLRTSPQWLGPLVEDLIHAHAVLSIE
PAML_A3         KFAVHHEEEVRQDEDEGILRQDRYPLRTSPQWIGPLVEDLISAHAALSIE
PAML_A4         KFAVHHEEEVRQDEDEGILRQDRYALRTSPQWIGPQLEDLLSAHKQISVE
                *:***** **  .:**********.** ****:** :.*:: **  :*:*

RgPAL           AGQSTTDNPLIDLENKMTHHGGAFMASSVGNTMEKTRLAVALMGKVSFTQ
MEGA_A1         AGQSTTDNPLIDVENKTTHHGGNFQAASVANTMEKTRLALAQMGKLSFTQ
MEGA_A3         LGNSTTDNPLIDVENKKIHHGGNFQAMSVTNAMEKTRLALQQMGKLSFTQ
PAML_A1         AGQSTTDNPLIDVENKTTHHGGNFQAASVANTMEKTRLALAQMGKLSFTQ
PAML_A2         AGNSTTDNPLIDVENKTVHHGGNFQAASVSNTMEKTRLALAQMGKLSFTQ
PAML_A3         LGNSTTDNPLIDVENKKIHHGGNFQAMSVTNAMEKTRLALQQMGKLSFTQ
PAML_A4         LGNSTTDNPLIDVENKKIHHGGNFQAMSVTNAMEKTRLALQHLGKLSFAQ
                 *:*********:***  **** * * ** *:*******:  :**:**:*

RgPAL           LTEMLNAGMNRALPSCLAAEDPSLSYHCKGLDIAAAAYTSELGHLANPVS
MEGA_A1         LTEMLNAGMNRGLPSCLAAEDPSLSYHCKGLDIAAAAYTSELGHLANPVT
MEGA_A3         MTELVNCSMNRGLPSCLAAEDPSTSYHCKGLDIAAAAYTSELGFLANPVS
PAML_A1         LTEMLNAGMNRGLPSCLAAEDPSLSYHCKGLDIAAAAYTSELGHLANPVS
PAML_A2         LTEMLNASMNRGLPSCLAAEDPSLSYHCKGLDIAAAAYTSELGYLANPVS
PAML_A3         MTELINCSMNRGLPSCLAAEDPSTSYHCKGLDIAAAAYTSELGFLANPVS
PAML_A4         MTELINCSMNRGLPSCLAGDEPSTNYHCKGLDIAAAAYTSELGFLANPVS
                :**::*..***.******.::** .******************.*****:

RgPAL           THVQPAEMGNQAINSLALISARRTAEANDVLSLLLATHLYCVLQAVDLRA
MEGA_A1         THVQPAEMGNQAVNSLALISARRTAEANDVLSLLLATHLYCVLQAIDLRA
MEGA_A3         THVQPAEMSNQAVNSLALISARKTVEANDVLSMLMATHLYCACQALDLRA
PAML_A1         THVQPAEMGNQAVNSLALISARRTAEANDVLSLLLATHLYCVLQAVDLRA
PAML_A2         THVQPAEMGNQAVNSLALISARRTAEANDVLSLLLATHLYCALQAVDLRA
PAML_A3         THVQPAEMSNQAVNSLALISARKTAEANDVLSLLLATHLYCACQALDLRA
PAML_A4         THVQSAEMHNQAVNSLALISARKTIEAVEVLSLLMSSHLYCACQALDLRA
                ****.*** ***:*********:* ** :***:*:::****. **:****

RgPAL           MEFEHTKAFEPMVTELLKQHFGALATAE-----VEDKVRKSIYKRLQQNN
MEGA_A1         MEFEFKKQFEPMIVELLKQHFGSLMTGENL-RELVEKVKKTLWKRLEQNN
MEGA_A3         LEFTFRKQFDPMILEMLKQHFGSFLTSEEL-KELTTKVTKTIWRRLEQTS
PAML_A1         MEFEFKKQFEPMIVELLKQHFGSLMTAENLRDELVDKVRKSLYKRLEQNN
PAML_A2         LEFEFKKQFEPMIVELLTQHFGSLLTSETLRDELVTKVKKSLYKRLEQNS
PAML_A3         LEFTFRKQFDPMIVELLTQHFGSFLTSEA--EDLATKVTKALYKRLEQHS
PAML_A4         LDLTFRKQLEAMLKELLTTHFGSFLTAEA--EDLSTKVAKALYKRLEQHS
                ::: . * ::.*: *:*. ***:: *.*     :  ** *::::**:* .
```

```
RgPAL           SYDLEQRWHDTFSVATGAVVEALAGQEVSLASLNAWKVACAEKAIALTRS
MEGA_A1         SYDLVPRWHDAFSFATGTVVEALASTELSLAALNAWKVACAEKAISLTRQ
MEGA_A3         SMDLVPRWHDAFNHVTSIVVEALSTSENPLPKINQWKKECAEQAVTLTRQ
PAML_A1         SYDLVPRWHDAFSFATGAVVEALASTSLSLAALNAWKVAAAEKAIALTRQ
PAML_A2         SYDLVPRWHDAFSFATGVVVEALASSSLSLAALNQWKVAAAEKAIALTRQ
PAML_A3         SVDLVPRWHDAFSHATGVVVEALASSELSLAALNQWKAAAAEQAVTLTRQ
PAML_A4         SMDLAPRFQDAFKHATGVIVEYLASSATSLASISSWRAAAAEQATKLYRQ
                * **  *::*:*. .*. :** *:    .*. :. *:  .**:*  * *.

RgPAL           VRDSFWAAPSSSSPALKYLSPRTRVLYSFVREEVGVKARRGDVYLGKQEV
MEGA_A1         VRETFWSAPSTSSPALSYLSPRTRILYSFVREELGVKARRGDVFLGKQEV
MEGA_A3         VRESFWSAPSTSSPAVEYLGPRTKALYSFVREELGVKARRGDVFLGKQEA
PAML_A1         VRETFWSAPSSSSPALSYLSPRTRILYSFVREELGVKARRGDVFLGKQEV
PAML_A2         VRETFWSAPSSSSPALSYLSPRTRILYSFVREELGVKARRGDVFLGKQEA
PAML_A3         VRESFWSAPSSSSPAVEYLGPRTKALYSFVREELGVKARRGDVFLGKQEA
PAML_A4         VRESFFSDPRSSAPAAEYLGPKTKALYEFVRQELGVKARRGDVALGRQEA
                **::*:: * :*:** .**.*:*: **.***:*:********* **:**.

RgPAL           TIGTNVSRIYEAIKSGRIAPVLVKMMA
MEGA_A1         TIGSNVSKIYEAIKSGRINKVLVKMMA
MEGA_A3         TIGSNVSKIYEAIKSGRINKVLVDMMA
PAML_A1         TIGSNVSKIYEAIKSGRINKVLVKMMA
PAML_A2         TIGSNVSKIYEAIKSGRINKVLVDMMA
PAML_A3         TIGSNVSKIYEAIKSGRINKVLVDMMA
PAML_A4         TIGSNVSKIYEAIRSGRLMKVLVDMFA
                ***:***:*****:***:  ***.*:*
```

Supplementary Figure S3: Alignment of *Rg*PAL and all ancestral enzymes. Grey: alanine-serine-glycine triad that forms catalytic moiety MIO, cyan: all tryptophans.

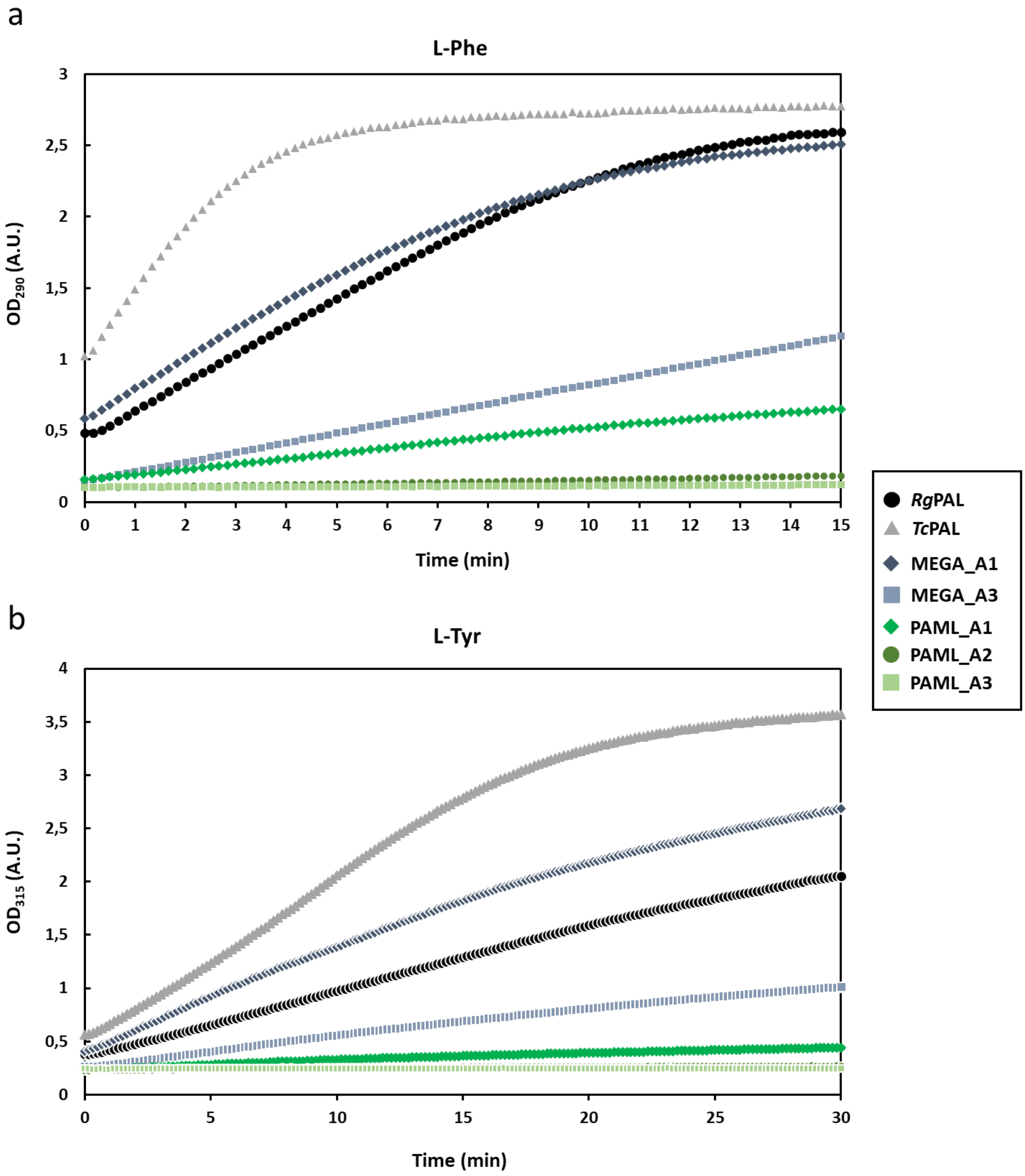

Supplementary Figure S4: Activity screen of all enzymes. All screens were performed with 10 mM substrate in a 50 mM Tris-HCl, 125 mM NaCl buffer (pH 8.5) at 37 °C. Enzyme in buffer without substrate was used as a control. **a)** Activity screen with substrate L-Phe. **b)** Activity screen with substrate L-Tyr. The results of these activity screens were only used as an indication of which enzymes to characterize in kinetic studies.

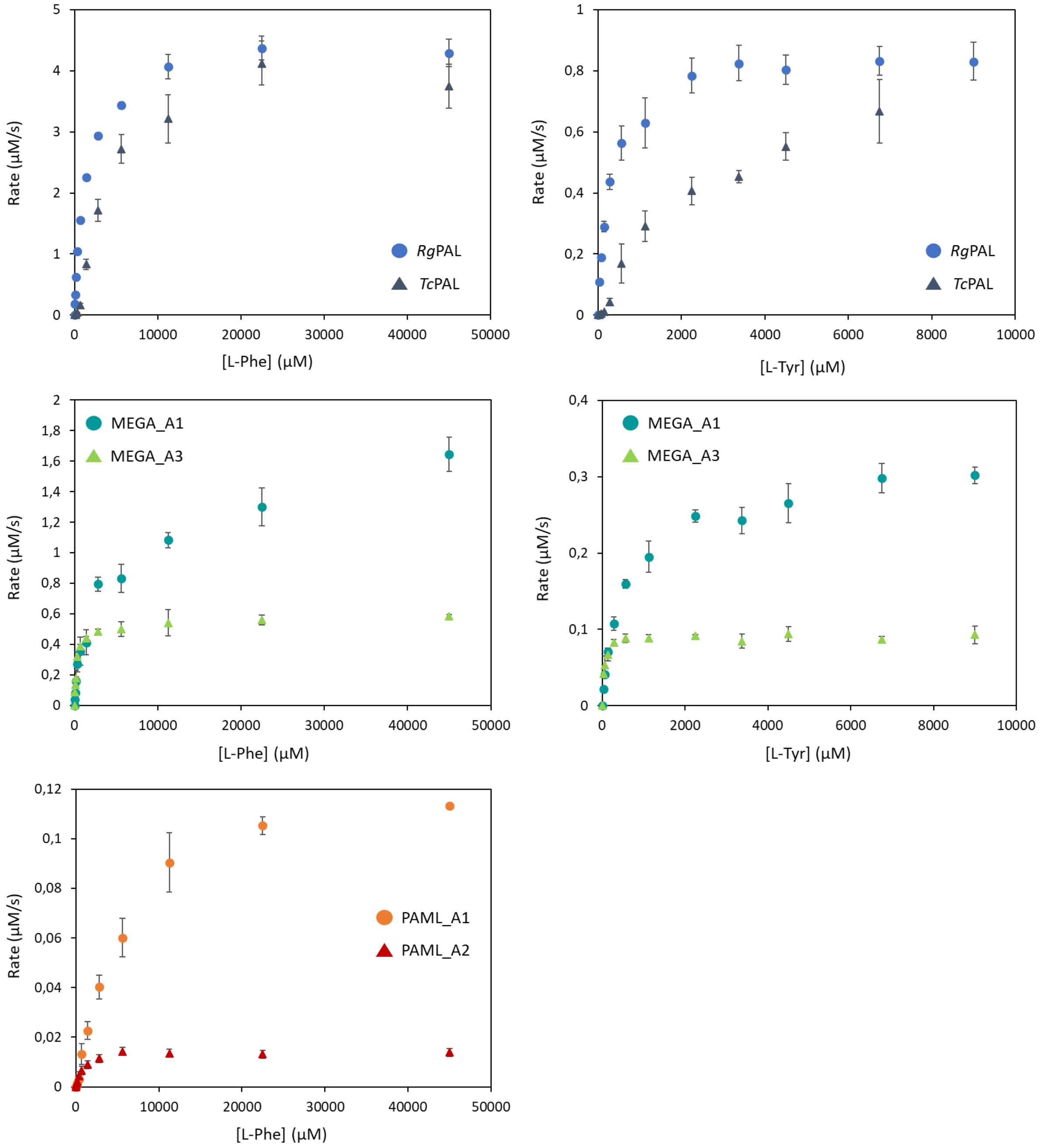

Supplementary Figure S5: Kinetic analyses of *Rg*PAL, *Tc*PAL and ancestral PAL/TAL with L-Phe and L-Tyr. All kinetic assays were performed in a 50 mM Tris-HCl buffer at pH 9.2 in 37 °C. Error bars show standard deviations with n=3.

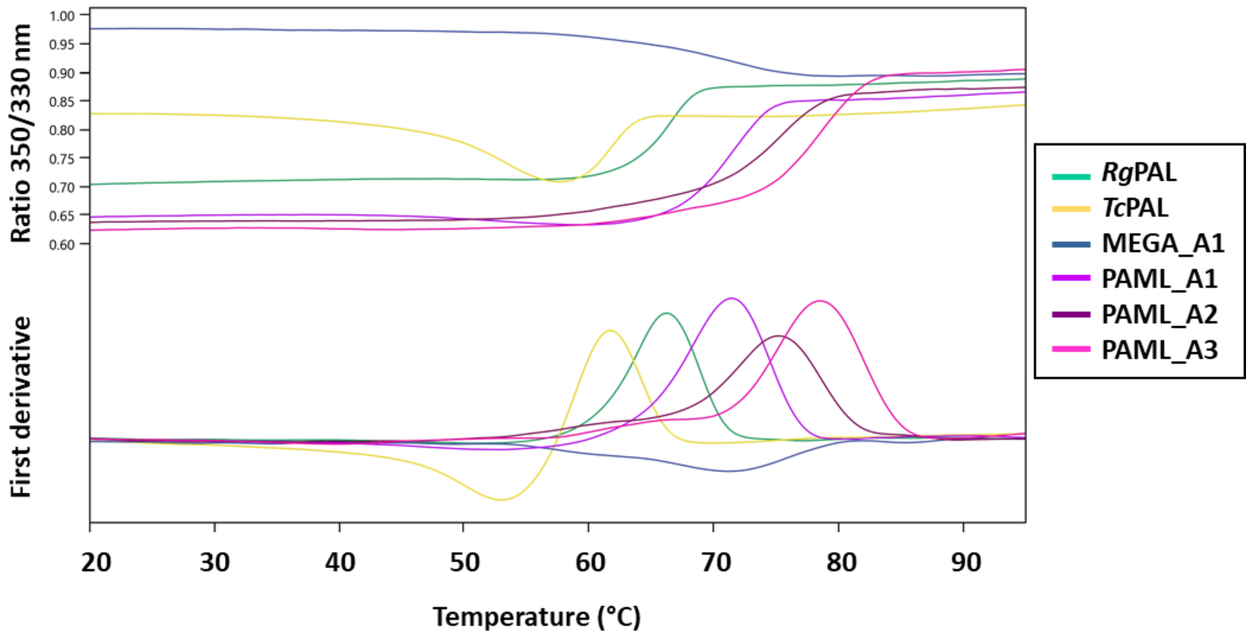

Supplementary Figure S6: Melting curves established by nanoDSF. The ratio of intrinsic tryptophan fluorescence at 350 over 330 nm was monitored while heating the samples with 1 degree per minute from 20 °C to 90 °C (top part of figure). The melting temperature is determined as the maximum of the first derivative of the ratio (bottom part of figure).

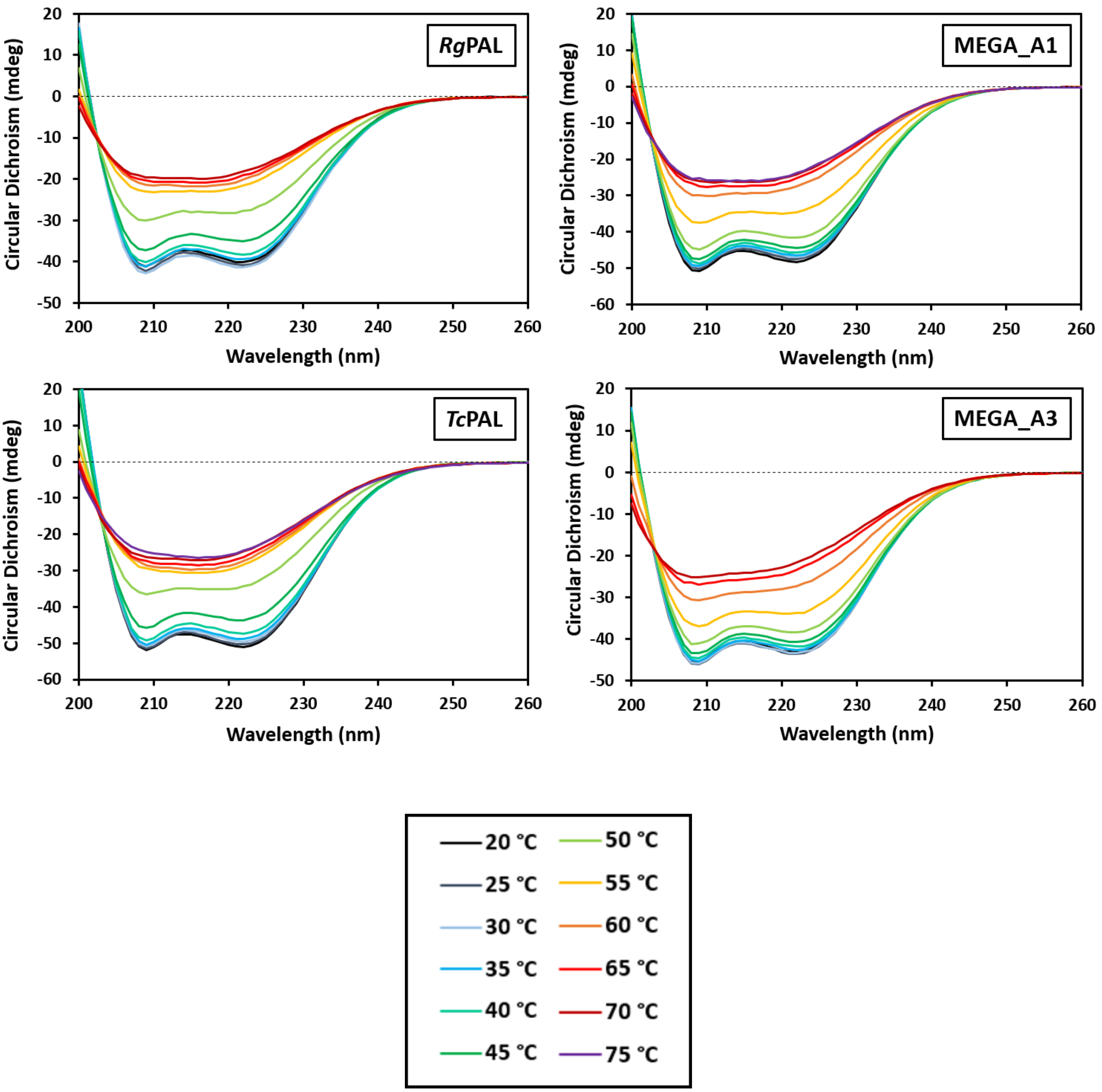

Supplementary Figure S7: Analysis of thermostability for *Rg*PAL, *Tc*PAL, MEGA_A1 and MEGA_A3 by circular dichroism spectroscopy. CD spectra were measured from 200-260 nm at one-degree intervals from 20-90 °C. Spectra are shown for every five-degree interval from 20-75 °C, since no additional changes were observed at higher temperatures and due to spectral overlap.

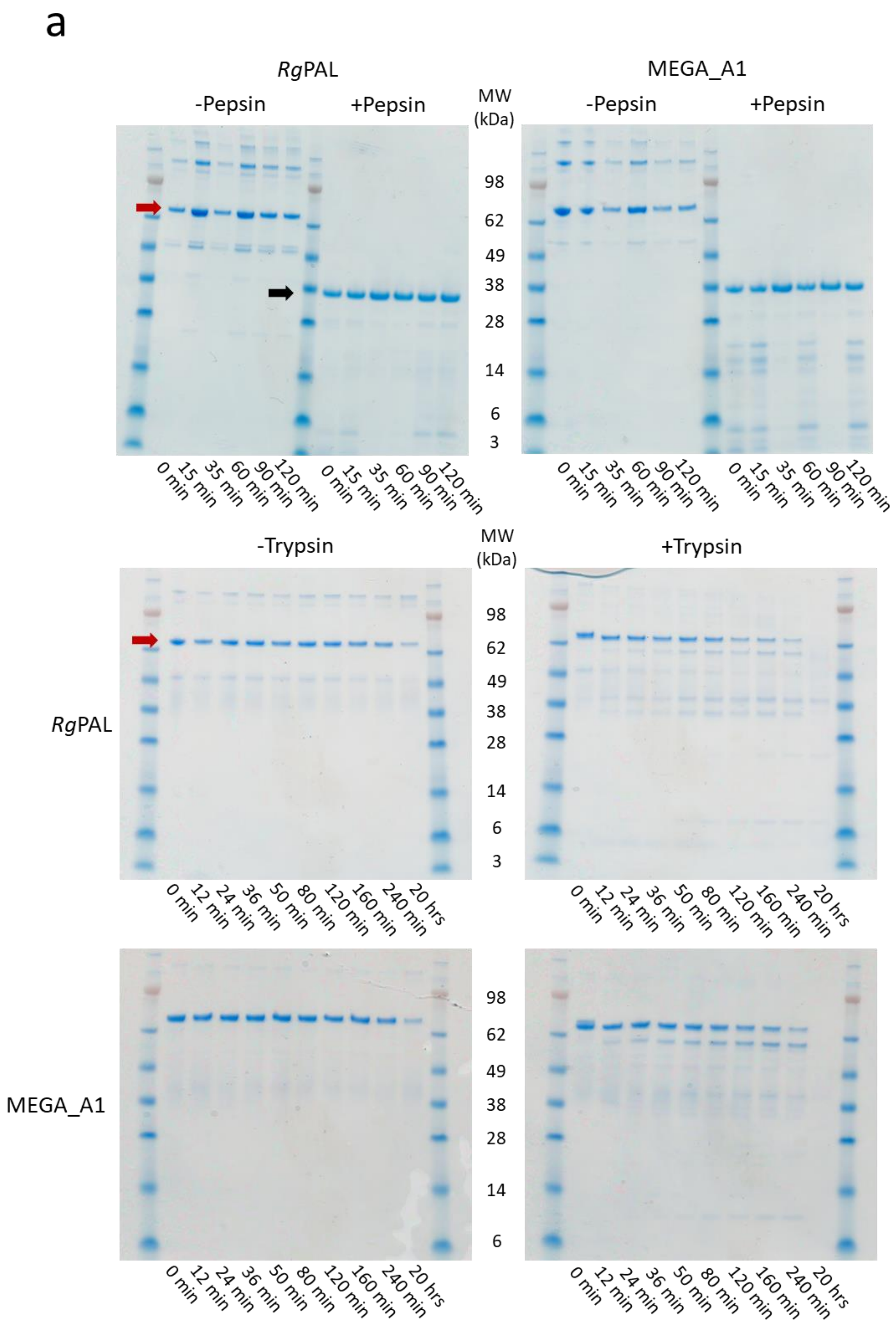

a
RgPAL
MEGA_A1
-Pepsin
+Pepsin
MW (kDa)
98
62
49
38
28
14
6
3
0 min
15 min
35 min
60 min
90 min
120 min
-Trypsin
+Trypsin
0 min
12 min
24 min
36 min
50 min
80 min
120 min
160 min
240 min
20 hrs

b

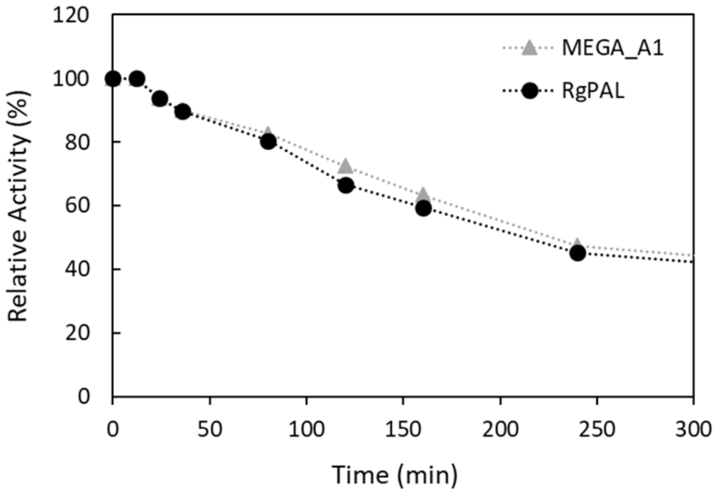

Supplementary Figure S8: Proteolytic stability. a) SDS-PAGE analysis of samples over time in samples +/- pepsin (top) and +/- trypsin (bottom). The band that represents PAL/TAL is indicated with a red arrow and disappeared completely in all pepsin samples. Pepsin is indicated by a black arrow. In samples with trypsin the PAL/TAL band becomes fainter over time, whereas other smaller bands appear (most likely degradation products). Due to the lower concentration of trypsin and a lower amount of protein loaded onto the gel, it is not visible on SDS-PAGE. All six gel images were cropped around the edges for display. The SeeBlue Plus2 pre-stained molecular weight marker is shown in all gels, however, the lowest band is missing from the MEGA_A1 trypsin gels because the program was run too long. Differences in band intensities for the +/- pepsin samples are likely due to inconsistent sampling (quantification of activity loss was based on kinetic measurements, see below). **b)** Activity analysis of *Rg*PAL and MEGA_A1 samples with trypsin over time, using L-Tyr as a substrate. Activity was lost completely after 20 hours. The sample taken after 50 minutes was only analyzed on SDS-PAGE because the temperature in the sample had not been adjusted to 37 °C for activity measurements and could not be included.

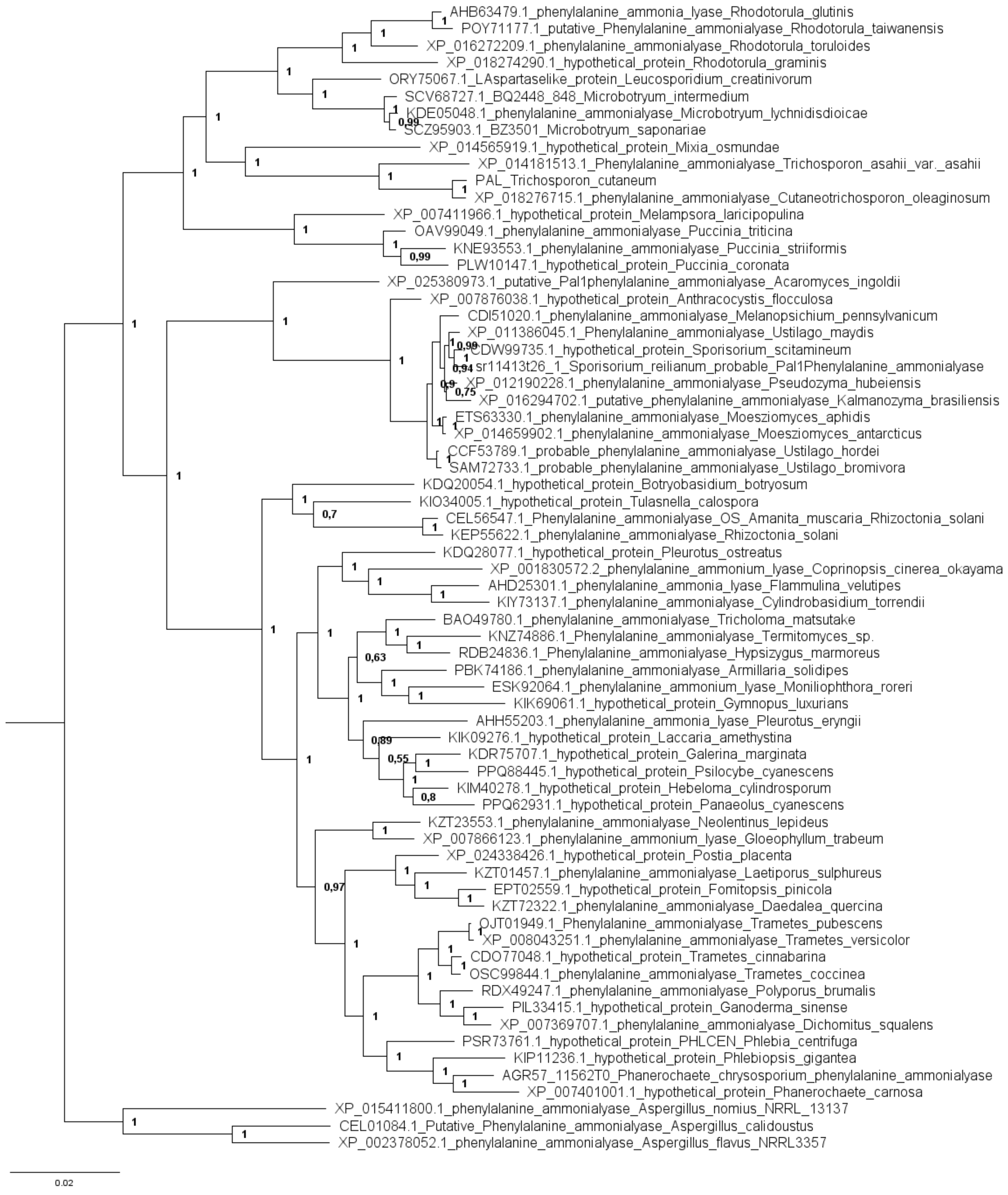

Supplementary Figure S9: Consensus tree from three chains in BAli-Phy. Three independent Markov Chain Monte Carlo chains were run in BAli-Phy[2] version 3.3 with the same set of sequences used to construct the Maximum Likelihood (ML) tree, yet in full length. Only two minor differences occur with respect to the ML tree - all within the clade of Agaricomycotina. The part in which the ancestors were reconstructed has high posterior probabilities.

REFERENCES

1. Scott, R. C. The genetic tyrosinemias. *Am. J. Med. Genet.* **142C**, 121–126 (2006).
2. Suchard, M. A. & Redelings, B. D. BAli-Phy: Simultaneous Bayesian inference of alignment and phylogeny. *Bioinformatics* **22**, 2047-2048 (2006).
